# Impact of epiretinal membrane peeling on steroid dependency in uveitic eyes: a retrospective analysis

**DOI:** 10.1186/s40942-025-00712-2

**Published:** 2025-07-29

**Authors:** Verena Schöneberger, Julia Schirrwagen, Claudia Brockmann, Thomas A. Fuchsluger, Friederike Schaub

**Affiliations:** 1 Department of Ophthalmology, University Medical Center Rostock, Doberaner Strasse 140, 18057 Rostock, Germany; 2 Department of Ophthalmology, Medical Faculty, University Hospital Düsseldorf, Heinrich Heine University Düsseldorf, Düsseldorf, Germany

**Keywords:** Uveitis, Epiretinal membrane, Membrane peeling, Cystoid macular edema, Steroids

## Abstract

**Background:**

Secondary epiretinal membranes (sERM) are common in uveitis and often associated with cystoid macular edema (CME), which increases the need for anti-inflammatory treatment. While surgical removal can improve anatomical and visual outcomes, its effect on intraocular inflammation and steroid requirement remains unclear. This study evaluates whether vitrectomy with ERM peeling can reduce the need for postoperative steroid therapy in uveitic eyes.

**Methods:**

This retrospective single-center study reviewed 67 eyes of 67 patients with history of uveitis who underwent sERM peeling between 11/2002 and 04/2023. Demographic data, uveitis classification (SUN), spectral domain optical coherence tomography (SD-OCT) findings, and pre-/postoperative steroid requirements were analyzed. Statistical significance testing was performed using a paired two-tailed t-test.

**Results:**

Of the 67 eyes, 50.7% were right eyes, and 65.7% of patients were female. Mean age at timepoint of surgery was 63.1 ± 13.6 years, with 53.7% phakic eyes. Uveitis was classified as anterior (17.9%), intermediate (44.8%), posterior (31.3%), and panuveitis (6.0%). Steroid therapy was reduced in 28.4% of patients, remained unchanged in 56.7%, and increased in 14.9%. Preoperatively, cystoid macular edema (CME) was present in 41.4% of the 58 available SD-OCT scans. Postoperatively, retinal thickness, macular volume, and total retinal volume decreased significantly (*p* < 0.001). Postoperative CME was found in 31.3% in first postoperative SD-OCT and was newly observed in 6.0%, while 62.7% showed no CME.

**Conclusions:**

ERM peeling in uveitic eyes does not guarantee functional improvement or a consistent reduction in steroid dependency. While approximately one-third of patients benefited from reduced steroid use—particularly those with preoperative CME—the majority showed no change, and a subset required intensified therapy due to postoperative inflammation or CME recurrence. Careful patient selection remains essential.

## Background

An epiretinal membrane (ERM) is a pathological layer overlying the inner retinal surface of the central retina as a result of myofibroblastic cell proliferation [1]. Due to its contractile properties, the ERM can distort the underlying retinal architecture, leading to metamorphopsia and reduced visual acuity. While idiopathic ERMs have a prevalence of approximately 9.1% [2], the prevalence of secondary ERM in patients with uveitis is higher - up to 41% - with intermediate uveitis cases reaching an incidence as high as 57% [3]. 

Cystoid macular edema (CME) is a common complication associated with ERM, further compromising vision and correlating with a poorer visual prognosis [1, 4]. It is important to distinguish CME from epiretinal membrane-foveoschisis, a condition characterized by a traction-induced separation of the foveal inner layers from the outer nuclear and plexiform layers [5]. 

The standard surgical treatment for ERM, including those of idiopathic origin, is pars plana vitrectomy (PPV) with ERM peeling. However, the application of this surgical approach in uveitic cases remains controversial. In uveitis, the secondary ERM is often associated with chronic inflammation, which complicates decisions regarding the optimal timing for surgery and the management of pre- and postoperative therapy. Despite an increasing number of studies investigating this approach, data specifically addressing surgical outcomes in uveitic ERM is limited. To date, only six studies have included more than ten eyes, with a cumulative total of 146 eyes analyzed [6–11]. 

Although the overall cohort in these studies shows a modest improvement in visual acuity after surgery, outcomes varied with some patients experiencing unfavorable results [8]. Besides visual acuity Tanawade et al. demonstrated that PPV with ERM peeling in uveitic eyes could not only improve macular traction but also contribute to the resolution of CME [8]. Similarly, Cristescu et al. observed a reduction in CME occurrence postoperatively, although some studies have reported persistent CME in certain cases [6, 10]. 

Given the frequent use of steroids in managing uveitis—whether to alleviate anterior chamber or vitreous inflammation or to treat CME—the potential of surgical ERM peeling to reduce postoperative steroid dependency warrants investigation. This study aims to address the following question: Can peeling of a uveitic secondary ERM reduce the necessity for or amount of glucocorticoids postoperatively?

## Methods

In this retrospective study, we reviewed records of 100 consecutive patients who underwent PPV with peeling of uveitic sERM between November 2002 and April 2023 at the Department of Ophthalmology, Rostock University Medical Center, Rostock.

The study was approved by the local Institutional Review Board (A 2022 − 0124) and was conducted in adherence to the tenets of the Declaration of Helsinki.

### Clinical information and collected data of recipients

Demographic and clinical data collected included etiology of uveitis, gender, age at time of sERM peeling, affected eye, lens status, best corrected visual acuity (BCVA) tested with decimal charts before and in follow-up examinations after surgery, as well as the type of surgery performed. Furthermore, collected data included therapy for uveitis. A **reduction** in therapy was defined as either discontinuation of the complete preoperative steroid regimen or a switch to a less intensive treatment. The hierarchy of treatment intensity was defined as: local < intravitreal (IVT) < systemic therapy, with systemic steroids considered equivalent to steroid-sparing agents. Within the IVT group, potency was ranked as follows: triamcinolone < dexamethasone implant < fluocinolone acetonide implant. Conversely, an **increase** in therapy was defined as the need to escalate to a more intensive treatment category or to a stronger agent within the same group. The preoperative steroid therapy was compared with the entire postoperative available follow-up period. The most potent steroid therapy was taken into account, even if it was only prescribed temporarily due to an inflammatory episode.

### Inclusion and exclusion criteria

**Fig. 1:**
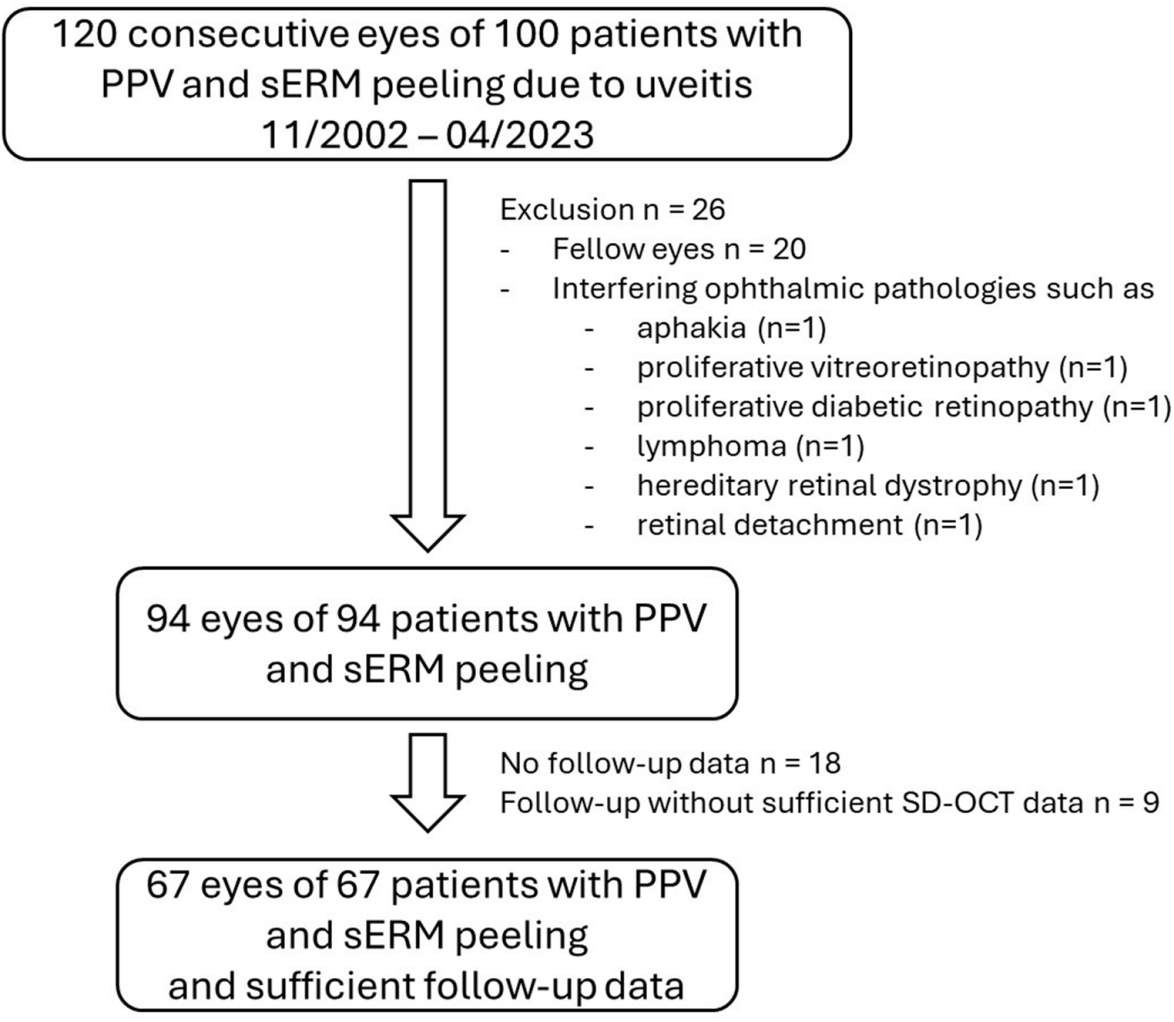
Flow chart of the cases included in the evaluation. Abbreviations: PPV - pars plana vitrectomy; SD-OCT - spectral domain optical coherence tomography; sERM – secondary epiretinal membrane

Records of all consecutive eyes with uveitis anterior, intermedia, posterior or panuveitis that had undergone PPV with sERM peeling were reviewed. Affected fellow eyes (*n* = 20) were randomly excluded from a total of 120 eyes with uveitic sERM to avoid distorting the statistical analysis with systemic and often disease-specific influences. Further exclusion was applied in case of interfering ophthalmic pathologies, which could also cause inflammation (like aphakia, proliferative vitreoretinopathy, proliferative diabetic retinopathy, lymphoma). Furthermore, missing follow-up data or insufficient follow-up OCTs served as exclusion criteria (see Flow chart in Fig. 1).

### Surgical procedure

Eyes underwent standard three-port complete vitrectomy (20 or 23 gauge) with ILM peeling using vital dye (Twin Blue; Alchimia, Italy or Brilliant Peel Dual Dye^®^; Geuder, Germany). If necessary, combined cataract extraction was performed by standard 2.8 mm clear cornea bimanual phacoemulsification followed by in-the-bag lens implantation. Surgery was performed by one of five experienced vitreo-retinal surgeons.

The retina was inspected for breaks or degeneration and treated if necessary. For endotamponade, filtered atmospheric air, balanced salt solution (BSS) or sulphur hexafluoride (SF6 25%) were used as needed, one case with a tractive detachment required silicone oil (Siluron 5000^®^; Geuder, Germany). Depending on the surgeon’s decision, subconjunctival, intravitreal or intravenous corticosteroids were applied in a non-standardised perioperative management process. All patients received routine postoperative treatment, which included topical antibiotic for one week and prednisolone acetate dops administered at least five times a day, with tapering doses over a period of two to four weeks.

### SD-OCT examinations and measurements

All eyes were examined using spectral domain optical coherence tomography (SD-OCT; SPECTRALIS^®^ HRA + OCT, Heidelberg Engineering GmbH, Heidelberg, Germany). A standard raster scan protocol was employed, consisting of 19 sections (512 A-scans per section) covering a 30° × 20° field of view.

**Fig. 2:**
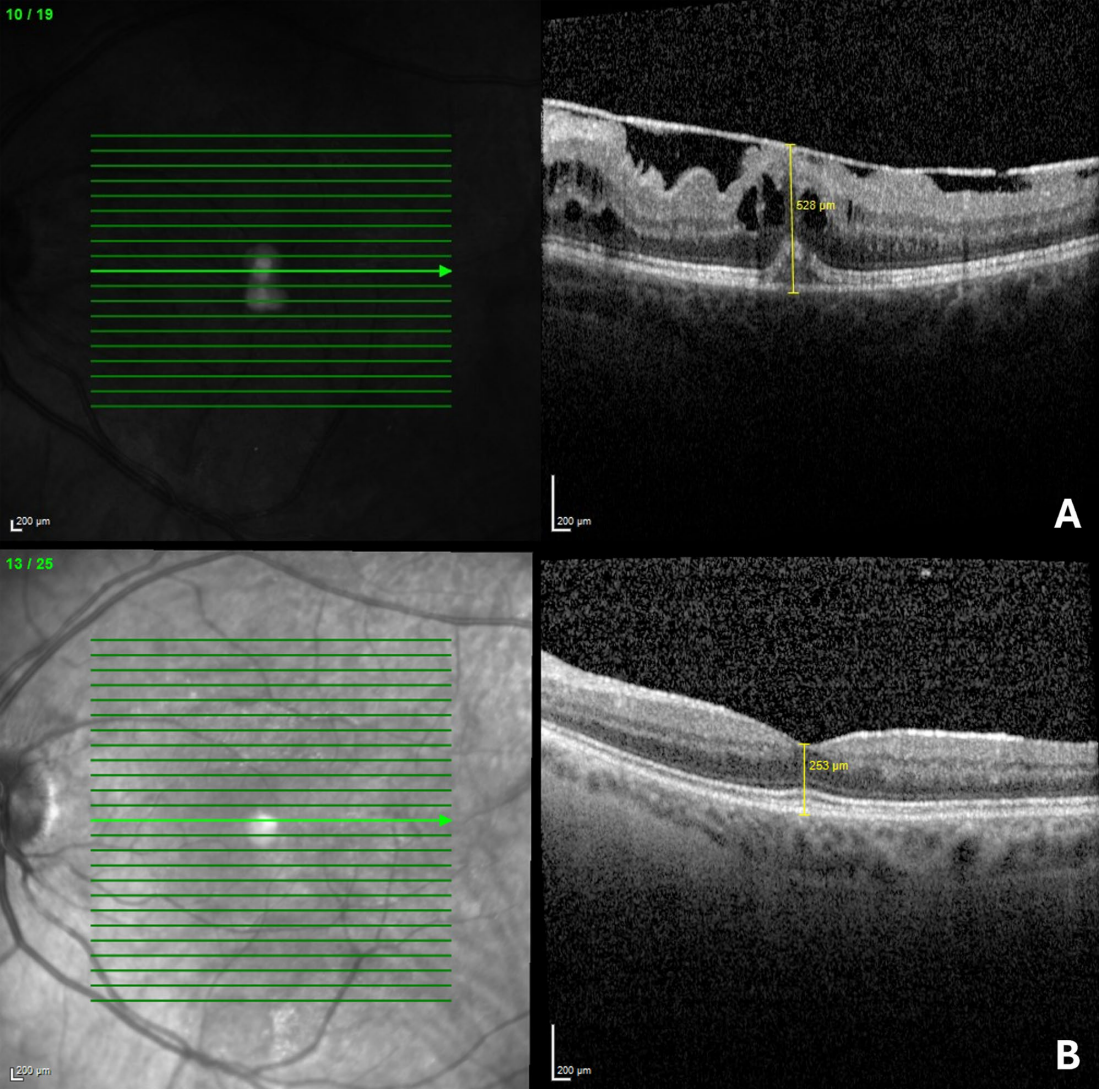
Spectral Domain Optical Coherence Tomography (SD-OCT) of a left eye. (**A**) Preoperative SD-OCT of a 46-year-old woman diagnosed with non-infectious posterior uveitis. The SD-OCT reveals focal traction in an epiretinal membrane (ERM), characterized by small, focal adhesions with hyporeflective spaces between the ERM and the internal limiting membrane (ILM), typical for uveitic ERM. Best-corrected visual acuity (BCVA) is 0.3 logMAR. Central retinal thickness (CRT) is 528 μm. (**B**) Five-year postoperative SD-OCT without ERM recurrence or macular edema and BCVA of -0.1 logMAR

Preoperative and postoperative anatomical characteristics, such as central retinal thickness (CRT) and central macular volume or total macular volume measured within the ETDRS grid, were recorded. Additionally, the presence or absence of perifoveal extracellular cystoid spaces in OCT scans was assessed (see Fig. 2).

### Statistical analyses

Descriptive statistics were conducted using SPSS (version 29.0 for Windows; SPSS, Inc., Chicago, IL). Best Corrected Visual Acuity (BCVA) values were converted to the logarithm of the Minimum Angle of Resolution (logMAR). Statistical significance testing for interval-scaled parameters was conducted using Student’s t-test, depending on the normality of the data distribution. A p-value of < 0.05 was considered statistically significant.

## Results

### Preoperative characteristics

Of the 67 eyes included in this study, 50.7% were right eyes, and 65.7% of patients were female. At the time of surgery, the average patient age was 63.1 ± 13.6 years (ranging from 22 to 88 years), and 53.7% (*n* = 36) were phakic. Preoperative best-corrected visual acuity (BCVA) was 0.45 ± 0.27 logMAR. According to the SUN classification, uveitis was categorized as anterior in 17.9% (*n* = 12), intermediate in 44.8% (*n* = 30), posterior in 31.3% (*n* = 21), and panuveitis in 6.0% (*n* = 4). Seven cases were diagnosed with infectious uveitis (toxoplasmosis, yersiniosis, borreliosis, lues, chlamydia).

Regarding steroid therapy, 53.8% (*n* = 32) eyes did not receive any steroids immediately prior to sERM peeling. The remaining eyes underwent various treatment regimens, including local, systemic, intravitreal and steroid-saving therapies, either alone or in combination. A detailed overview is provided in Table 1.

Preoperative spectral-domain optical coherence tomography (SD-OCT) was available for 58 eyes, of which 24 (41.4%) showed CME in addition to sERM. Among these, 83.3% (*n* = 20) received steroid treatment in the preoperative period. The specific treatment modalities are also summarized in Table 1.

### Surgical characteristics

None of the eyes presented with inflammatory activity such as cells in the anterior chamber and vitreous at least three months prior to and at timepoint of surgery. PPV was combined with ERM peeling and in 95.5% with ILM peeling. In one case, combined cataract surgery was performed. Endotamponade of choice was filtered air in 62.7% (*n* = 42) of cases, followed by SF6 25% in 25.4% (*n* = 17), BSS in 10.4% (*n* = 7) and Siluron 5000 in one eye. Subconjunctival dexamethasone was administered at the end of surgery in 59 of 67 eyes and intravitreal triamcinolone in the remaining 8 eyes. In addition, 23 patients received intravenous prednisone 250 mg during surgery.

### Postoperative follow-up

The median follow-up time was 31.0 months (Q1 10.0; Q3 51.0; range 0-185 months). Preoperative best corrected visual acuity (BCVA) was 0.45 ± 0.27 logMAR and decreased to 0.57 ± 0.72 logMAR at the last follow-up (*p* < 0.001).

The first follow-up examination in our clinic was performed within the first 3 months after surgery in 42 cases, within the first 6 months after surgery in a total of 52 cases, within a year in 56 cases and more than one year after surgery in 11 cases. Overall, at the first follow-up examination the central retinal thickness (CRT) decreased significantly from 423.05 ± 132.81 μm preoperatively to 360.94 ± 148.00 μm postoperatively (*p* < 0.001). Similarly, central macular volume decreased from 0.35 ± 0.10 mm³ to 0.31 ± 0.10 mm³ and total retinal volume within the ETDRS grid decreased from 9.90 ± 1.98 mm³ to 9.08 ± 1.43 mm³, both with a statistically significant reduction (*p* < 0.001) compared to preoperative values. Postoperative persistent CME was seen in 21 of 67 eyes on the first postoperative SD-OCT and new CME was seen in 4 eyes. No CME was found in 42 eyes (62.7%).

Regarding steroid dependency after surgery compared to before surgery, there were more eyes without steroid or steroid-saving treatment after surgery with 62.7% than before with 46.3%. The heterogeneous therapy with local, systemic or intravitreal or steroid-sparing therapy is listed in Table 1.

**Table 1:** Preoperative and postoperative steroid treatment during follow-up period in all eyes and subgroup with CME

Steroid treatment	Percent of all eyes before surgery *N* = 67 (number)	Percent of all eyes after surgery *N* = 67 (number)	Percent of eyes with CME before surgery *N* = 24 (number)	Percent of eyes with preoperative CME after surgery *N* = 24 (number)
none	46.3% (31)	62.7% (42)	16.7% (4)	66.7% (16)
	*p* = 0.005*		*p* = 0.052*	
Local steroids (1 to 5 times a day)	7.5% (5)	1.5% (1)	16.7% (4)	4.2% (1)
Systemic steroids (5 to 20 mg)	4.5% (3)	4.5% (3)	8.3% (2)	0
Local + systemic steroids	1.5% (1)	1.5% (1)	0	0
Steroid saving therapy *ciclosporin 400 mg daily, methotrexate 7.5 mg weekly, adalimumab 40 mg every 2nd week	4.6% (3)	3.0% (2)	4.2% (1)	8.3% (2)
Triamcinolone 4 mg intravitreal	7.5% (5)	6.0% (4)	12.5% (3)	0
Dexamethasone 700 µg intravitreal	22.4% (15)	12.0% (8)	29.2% (7)	20.8% (5)
	*p* = 0.181*		*p* = 0.608*	
Fluocinolonacetonid 190 µg intravitreal	-	4.5% (3)	0	0
Systemic steroids + Dexamethasone intravitreal	4.5% (3)	1.5% (1)	8.3% (2)	0
Steroid saving therapy + Dexamethasone intravitreal	1.5% (1)	3.0% (2)	4.2% (1)	0

preoperative and postoperative steroid treatment during follow-up period (median 31.0 months) of 67 eyes with secondary uveitic epiretinal membrane and the subgroup of 24 eyes with additional cystoid macular edema (CME). *Fisher’s Exact test

Comparing pre- and post-operative therapy on an individual basis, 56.7% (38) of patients needed a comparable therapy regime, 28.4% [19] had reduced therapy dependency and 14.9% [10] had increased therapy dependence after PPV with sERM peeling.

Focusing on reasons for increased steroid treatment, 5 eyes needed increased treatment due to reactivation of uveitis with cells in the anterior chamber or vitreous. 4 of the remaining 5 eyes were treated with higher doses of steroids because of recurrent or new CME. 2 of these 4 cases with CME had additional cataract surgery a few weeks to months before implicating coexisting components of postoperative macular edema. One eye had a retinal detachment due to proliferative vitreoretinopathy and therefore needed more steroids.

Subgroup analysis regarding SUN-classification showed no difference in means of steroid need after sERM peeling.

Further **subgroup analysis** was performed on **reactivation of inflammation** with anterior chamber and/or vitreous flare, recurrence of CME and the influence of cataract surgery in phakic eyes for steroid treatment:

- *Uveitis reactivation*.

In 12 eyes, there was a reactivation of uveitis with inflammatory cells in anterior chamber or vitreous. Median time was 31.0 months (Q1 8.0; Q3 87.0). Of these, 5 eyes received dexamethasone, and one received intravitreal triamcinolone. Out of the 12 eyes, 2 needed reduced glucocorticoid treatment postoperatively, 4 remained unchanged and in 6 eyes treatment increased. In this cohort, 50% (6/12) of cases are presented with persistence of CME on SD-OCT. Three eyes in the subgroup with reactivation of uveitis suffered a recurrence of ERM with 25.0% (3/12). In contrast, in the non-reactivation group only 27.8% (15/54) showed persistence of CME and in 3.7% (2/51) ERM recurrence. In 1 case, the fellow eye was reactivated with increased systemic steroid treatment.

- *Cystoid macular edema (CME).*

Of the 24 patients with preoperative CME (41.4%), 14 had persistent CME. In 8 cases the steroid treatment was similar, in 16 cases it was reduced (66.7%) (Table 1). Overall, 10 out of 24 eyes (41.7%) showed postoperative resolution of CME. However, it should be noted that half of these eyes had a recurrence of CME during the postoperative follow-up period. Steroid treatment in the group with resolved CME after PPV remained similar in 5 cases, decreased in 4 cases and increased in one case due to PVR reaction.

On the first postoperative SD-OCT, cystoid macular edema (CME) was present in 25 of 65 eyes (37.3%). Analysis showed a higher CRT preoperative as a risk factor for developing a new CME postoperative with CRT 478.75 ± 133.76 μm preoperatively to the group without postoperative CME development with 384.30 ± 121.95 μm (*p* = 0.006).

**Fig. 3:**
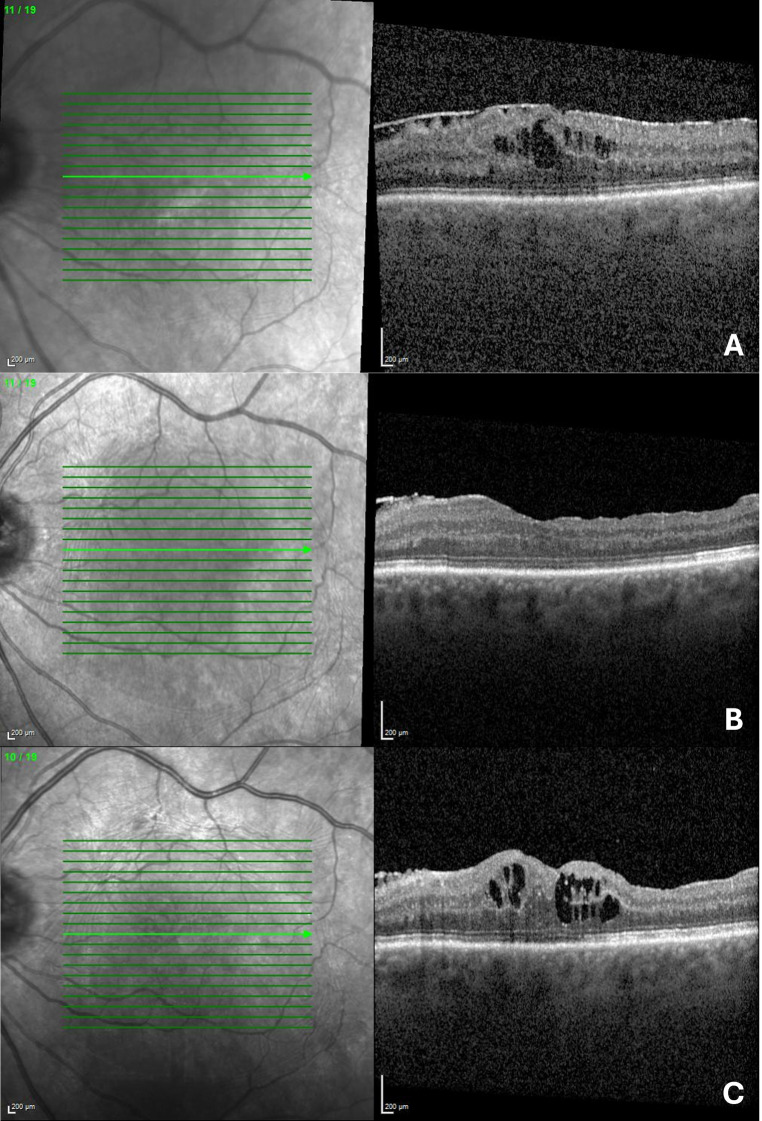
Spectral Domain Optical Coherence Tomography (SD-OCT) of a left eye of a 68-year-old man diagnosed with intermediate uveitis. (**A**) Preoperative SD-OCT: Shows an epiretinal membrane (ERM) with hyporeflective spaces between the ERM and the internal limiting membrane (ILM). The patient received two dexamethasone implants preoperatively. Best-corrected visual acuity (BCVA) was 0.8 logMAR. (**B**) Three months postoperative SD-OCT: No signs of cystoid macular edema (CME) are present, with improved BCVA at 0.3 logMAR. (**C**) One-year postoperative SD-OCT: Development of CME is observed, accompanied by a decline in visual acuity to 0.4 logMAR. Decision for a dexamethasone implant was made

In the further follow-up period in 10 eyes (14.9%) a recurrence of CME was present with a median of 18.0 months (Q1 4.5; Q3 35.0) and CME newly developed in 9 eyes (13.4%) with a median time of 5.5 months (Q1 3.0; Q3 7.0) (Fig. 3).

Regarding CME, cases were divided into resolved, persistent, and newly developed after PPV according to the SUN classification in Table 2. As preoperative OCT scans were only available in 58 cases, this comparison refers to this group.

**Table 2:** Cystoid macular edema after vitrectomy with ERM peeling

Uveitis SUN-classification	Anterior (10)	Intermedia (24)	Posterior (20)	Panuveitis (4)
No CME Pre-/Post-PPV	30.0% (3)	62.5% (15)	60.0% (12)	(0)
CME outcome after surgery with follow-up (median 31.0 months)				
Resolved CME after PPV	(0)	20.8% (5)	15.0% (3)	50.0% (2)
Resolved CME without recurrence	(0)	12.5% (3)	5.0% (1)	25.0% (1)
Persistent CME	40.0% (4)	16.7% (4)	20.0% (4)	50.0% (2)
Newly developed CME post-PPV	30.0% (3)	(0)	5.0% (1)	(0)

CME with resolved, persistent, new after pars plana vitrectomy and recurrence CME in follow-up period after pars plana vitrectomy according to SUN-classification in 58 eyes with pre- and postoperative SD-OCT. We defined ‘resolved’ CME as the absence of CME at first postoperative examination following hospitalisation, usually six weeks after PPV. A new CME could have been diagnosed in any postoperative phase during the follow-up period. (CME – cystoid macular edema, PPV – pars plana vitrectomy, SUN – Standardization of Uveitis Nomenclature)

- *Cataract surgery*.

Of 36 phakic eyes, 25 eyes (69.4%) underwent cataract surgery after a median of 10.5 months (Q1 5.0; Q3 16.0; range 3–46 months). During the first 3 months after cataract surgery, we documented new or recurrent CME in 6 cases (24.0%). In two cases we saw an increased steroid need after cataract surgery.

After PPV 12 phakic eyes (33.3%) had persistent CME, and 3 out of 4 eyes developed new CME. In contrast in the pseudophakic group of 31 eyes, 9 eyes (29.0%) had persistent CME, and one eye had new CME after PPV.

## Discussion

Patients with uveitis represent a heterogeneous cohort with varying degrees of dependence on steroids or immunosuppressive therapy. Peeling of a sERM associated with CME theoretically reduces mechanical traction on the retina by removing the epiretinal tractional membrane and inflammatory stimuli by removing the vitreous matrix [6]. This could, in turn, lead to a decreased need for postoperative steroid therapy. In our results 16 out of 24 patients with preoperative CME needed reduced therapy (66.7%). However, in the overall cohort 28.4% of patients required less steroid therapy postoperatively, the majority (56.7%) showed no change, and 14.9% experienced an increase in steroid requirements. These findings highlight the complexity of uveitic pathology and the multifactorial nature of postoperative inflammation. Subgroup analysis regarding the SUN-classification showed no uveitis subgroup with greater benefit after sERM peeling.

Previous studies on ERM peeling in uveitic eyes have demonstrated mixed results. Tanawade et al. reported improvements in macular traction and CME resolution following vitrectomy with ERM peeling, supporting the notion that surgical intervention may aid in reducing inflammatory edema and reduce necessity of treatment with steroids [8]. Similarly, Cristescu et al. found a significant decrease in CME from 66% of eyes before to 28% after surgery, although 21% (6 eyes) experienced worsening of uveitis and required additional medication [6]. Our study aligns with these findings, showing that CME resolved postoperatively in 41.7% of cases, but recurrence remained an issue, occurring in approximately 20% of cases overall and in 75% of panuveitis cases. These results suggest that while selected patients can benefit from ERM peeling, it does not consistently prevent CME recurrence.

Another crucial aspect to consider is the influence of residual inflammation. Our results indicate that 12 eyes (17.9%) experienced uveitis reactivation postoperatively, necessitating increased steroid therapy. This finding emphasizes that despite surgical intervention, persistent intraocular inflammation may continue to disrupt the blood-retinal barrier, necessitating ongoing pharmacologic management. There was no difference in the use of 20- or 23-gauge PPV for uveitis reactivation or the increased need for steroid therapy. This was comparable to the results of the prospective controlled study by Scholz et al., which found no significant differences in laser flare measurements 3 weeks after PPV for macular hole or macular pucker [12]. By removing the inflammatory matrix in the vitreous, there could be a beneficial effect on levels of inflammatory cytokines and mediators of inflammation and therefore reduced need of anti-inflammatory treatment [13, 14]. A meta-analysis on PPV in uveitis (excluding ERM peeling) involving 300 eyes detected CME in 52% of eyes preoperatively and in 37% postoperatively. Moreover, the median use of oral corticosteroids dropped from 48 to 12% postoperatively [14]. In another study of 27 eyes undergoing PPV without macular intervention, systemic steroid and second-line immunosuppressive use was reduced by 26% at 12 months, with 87.5% of patients achieving resolution of macular edema [15]. 

Cataract surgery also played a role regarding CME persistence or recurrence. Of the phakic eyes in our study, 69.4% underwent cataract surgery after vitrectomy after median time of 10.5 months, with CME reactivation or new development occurring in six cases during the first 3 months after cataract surgery. The well-documented association between cataract extraction and postoperative pseudophakic macular edema (Irvine-Gass syndrome), as well as the role of intraocular surgery in disrupting the blood-aqueous barrier, underscores the need for careful perioperative management in these patients [16]. 

Not only cataract surgery, but also PPV itself could potentially lead to postoperative CME. In our study, postoperative persistent CME was seen in 21 of 67 eyes on the first postoperative SD-OCT and new CME was seen in 4 eyes. Of the 24 patients with preoperative SD-OCT and CME, 58.3% (*n* = 14) had persistent CME. In a study analyzing cases after PPV for vitreous floaters without manipulation of the macula, postoperative CME was seen in 5.5% of cases [17]. In another study pseudophakic eyes developed a new CME in 15.3% of cases within the first 2 months after PPV for different indications including RRD, ERM peeling or removal of silicone oil [18]. CME observed after ILM peeling for ERMs may derive more from glial degeneration rather than a true blood–retinal barrier disfunction [19]. 

Importantly, our study demonstrated a significant reduction in central retinal thickness (CRT), central macular volume, and total macular volume within the ETDRS grid postoperatively (*p* < 0.001), confirming successful anatomical restoration in accordance with other studies [6–9]. However, this morphological improvement did not translate into a significant functional benefit, as the best-corrected visual acuity (BCVA) did not improve postoperatively. This finding contrasts with other studies, which reported visual gains postoperatively, though the initial BCVA in those cohorts was worse compared to our study population [6, 7, 9, 20]. Notably, our results align with those of Tanawade et al., who also observed no significant visual improvement after ERM peeling in uveitic eyes after 6 months [8]. Peeling of sERM is only recommended in cases of ERM-related vision loss and when a worse initial visual acuity seems more favourable for postoperative visual improvement. This underscores that the decision for ERM peeling should not be based on the expectation of visual acuity improvement alone but rather on factors such as CME resolution and potential steroid-sparing effects.

A limitation of our study is its retrospective nature and the inclusion of data spanning over two decades, during which treatment strategies evolved. Another important point is that there was no standardized perioperative management process, with the decision on postoperative steroid treatment left to the surgeon. Follow-up time points and durations varied significantly, including the range of initial postoperative follow-up duration. Cases with persistent or recurrent CME were observed for a longer period, potentially introducing a bias. As it is not possible to clearly differentiate the origin of CME, i.e. whether it was caused by ERM and/or uveitis, all types of CME were included in our analysis. Cataract surgery performed after PPV without a standardized protocol or defined timing represents an additional confounding factor and should be acknowledged as an additional limitation. Nonetheless, this study represents one of the largest cohorts analyzed for uveitic ERM peeling, providing valuable insights into its effects on steroid dependency and CME management.

There are several factors which could influence steroid usage after ERM peeling and CME persistence. An important factor after ERM removal is active inflammation influencing the blood-ocular-barrier. Steroids or immunosuppressive therapy may still be necessary to control this inflammation. Probably, the beneficial effect of ERM peeling on reducing steroid requirements depends on how much inflammation and CME is attributable to the ERM. Even after successful ERM peeling a persisting uveitic or underlying autoimmune activity may necessitate ongoing steroid use.

## Conclusions

ERM peeling in uveitic eyes does not consistently result in functional improvement but may promote anatomical stabilization and CME resolution in selected cases. Postoperatively, 28.4% of patients experienced a reduction in steroid therapy, most notably among those with preoperative CME with 66.7%. However, overall 14.9% required intensified treatment due to inflammation reactivation or CME recurrence, while the majority (56.7%) showed no change. CME resolved in 41.7% of cases, yet recurrence remained a concern. Subgroup analysis based on SUN classification did not reveal any specific uveitis subtype with a clearly greater benefit. Given these variable outcomes, the indication for ERM peeling should be carefully weighed, particularly in the absence of progressive vision loss. Further prospective studies are warranted to identify patient subgroups most likely to benefit from this intervention.

## Data Availability

The data that support the findings of this study are available on request from the corresponding author. The data are not publicly available due to privacy or ethical restrictions.

## Abbreviations

BCVA: Best corrected visual acuity
BSS: Balanced salt solution
CME: Cystoid macular edema
CRT: Central retinal thickness
ETDRS: Early treatment diabetic retinopathy study
IL: Interleukin
ILM: Internal limiting membrane
logMAR: Logarithm of the Minimum Angle of Resolution
OCT: Optical coherence tomography
PPV: Pars plana vitrectomy
PVR: Proliferative vitreoretinopathy
sERM: Secondary epiretinal membrane
SF6: Sulfur hexafluoride
SD-OCT: Spectral domain optical coherence tomography
SUN: Standardization of uveitis nomenclature
